# Comparison of Exenatide and Metformin Monotherapy in Overweight/Obese Patients with Newly Diagnosed Type 2 Diabetes

**DOI:** 10.1155/2017/9401606

**Published:** 2017-11-20

**Authors:** Jia Liu, Yanjin Hu, Yuan Xu, Yumei Jia, Li Miao, Guang Wang

**Affiliations:** Department of Endocrinology, Beijing Chao-Yang Hospital, Capital Medical University, No. 8, Gongti South Road, Chaoyang District, Beijing 100020, China

## Abstract

**Aims:**

The present study assessed the therapeutic effect of exenatide and metformin as the initial therapy on overweight/obese patients with newly diagnosed type 2 diabetes (T2D).

**Methods:**

The prospective, nonrandomized, interventional study enrolled a total of 230 overweight or obese patients with newly diagnosed T2D who were administrated exenatide or metformin hydrochloride for 12 weeks.

**Results:**

224/230 patients, including 106 in the exenatide group and 118 in the metformin group, completed the 12-week treatment. Both exenatide and metformin significantly decreased the HbA1c levels in overweight/obese patients with newly diagnosed T2D (all *P* < 0.05). The reduction in HbA1c and the proportion of patients with HbA1c < 7.0% (53 mmol/mol) were higher in the exenatide group than in the metformin group (all *P* < 0.05). The exenatide treatment caused a greater decline in the body weight and BMI as compared to the metformin treatment (all *P* < 0.01). The exenatide treatment (*β* = 0.41, *P* < 0.01) and baseline HbA1c level (*β* = −0.84, *P* < 0.01) were independent influencing factors for the decrease in HbA1c level.

**Conclusions:**

For an initial therapy in overweight/obese patients with newly diagnosed T2D, exenatide causes a better glycemic control than metformin. This trial is registered with NCT03297879.

## 1. Introduction

Type 2 diabetes (T2D) is a common metabolic disease with high morbidity and mortality due to T2D-related complications [1]. Obesity is a major risk factor for T2D as it induces chronic inflammation, endoplasmic reticulum stress, mitochondrial dysfunction, and insulin resistance [2]. The impact on body weight is also considered a critical aspect of the clinical evaluation of hypoglycemic drugs. Several studies have confirmed that some hypoglycemic drugs like sulfonylureas, thiazolidinediones, or insulin tend to cause gain weight, while metformin leads to no change or mild decline in body weight [3]. Thus, metformin is frequently used as a first-choice therapy in overweight/obese T2D patients [3]. Glucagon-like peptide-1 (GLP-1) receptor agonist is a novel agent approved for treating T2D and has been demonstrated to induce significant weight loss in overweight/obese T2D patients [3–6].

However, it is yet unclear whether as initial treatment, GLP-1 receptor agonist contributes to an improved therapeutic efficacy than metformin in overweight/obese patients with newly diagnosed T2D. Exenatide is a classical drug of a GLP-1 receptor agonist. In the present study, we assessed the therapeutic effect of exenatide and metformin as the initial therapy on overweight/obese patients with newly diagnosed T2D.

## 2. Materials and Methods

### 2.1. Subjects

The present study was a prospective, nonrandomized, interventional study, performed between September 2013 and October 2015 at the Department of Endocrinology in Beijing Chao-Yang Hospital affiliated to Capital Medical University. We consecutively enrolled 230 drug-naïve, overweight, or obese patients with newly diagnosed T2D, who fulfilled the following inclusion criteria: (1) age 20 to <65 years, (2) body mass index (BMI) ≥24 kg/m^2^ [7], and (3) HbA1c ≥ 7% (53 mmol/mol). All of the patients have no diabetes antibodies and were diagnosed with T2D within the previous 3 months, according to the ADA diagnostic criteria [3]. None of the patients had been administered antidiabetic drugs or diet therapy before participation. Neither of the patients presented any history of pancreatitis, coronary artery disease, liver function impairment, renal function impairment, intestinal surgery, chronic hypoxic diseases (emphysema and cor pulmonale), infectious disease, hematological disease, systemic inflammatory disease, or cancer. Patients who were pregnant, possibly pregnant, or ingesting agents known to influence glucose or lipid metabolism were also excluded.

This study was conducted in accordance with the Declaration of Helsinki and approved by the Ethics Committee of Beijing Chao-Yang Hospital affiliated with Capital Medical University. All participants provided written informed consent.

### 2.2. Study Design

All participants were overweight or obese T2D patients, conforming to the indication of metformin or exenatide [3]. The patients were assigned to the exenatide group (110 patients) or metformin group (120 patients), respectively, according to their will. Exenatide was administrated with 5 *μ*g bid for 4 weeks and 10 *μ*g bid for 8 weeks. Metformin hydrochloride was initiated at a dose of 500 mg bid for 2 weeks and added to 2.0 g/day for 8 weeks. All patients were provided lifestyle counseling upon enrollment.

Patients were followed up every 4 weeks. At each visit, anthropomorphic parameters and adverse events were recorded. Height and weight were measured to the nearest 0.1 cm and 0.1 kg, respectively, by the same trained individuals. Minor hypoglycemia was defined as signs or symptoms confirmed by blood glucose < 3.9 mmol/L with a recovery after self-treatment. Major hypoglycemia was defined as signs or symptoms recovered after administration of carbohydrate, glucagon, or other resuscitative treatment or confirmed by blood glucose <3.0 mmol/L with the assistance of another individual.

Laboratory assays were performed at baseline. After 12 weeks of exenatide or metformin treatment, blood samples were obtained in the morning after overnight fasting and stored at −80°C. Total cholesterol (TC), low-density lipoprotein cholesterol (LDL-C), high-density lipoprotein cholesterol (HDL-C), and triglycerides (TG) were measured by colorimetric enzymatic assays, using an autoanalyzer (Hitachi 7170). FBG, fasting insulin (FINS), and HbA1c were measured at the Central Chemistry Laboratory of Beijing Chao-Yang Hospital affiliated with Capital Medical University. BMI was calculated as weight (kg)/height (m^2^). HOMA of insulin resistance (HOMA-IR) and homeostasis model assessment of *β*-cell function (HOMA-*β*) have been computed according to the following formulas: HOMA-IR = FINS (*μ*IU/mL) × FBG (mmol/L)/22.5 and HOMA-*β* = 20 × FINS (*μ*IU/mL)/FBG (mmol/L) − 3.5 [8].

### 2.3. Statistical Analysis

Data were analyzed using SPSS 17.0 (SPSS Inc., Chicago, IL, USA). Continuous data were expressed as mean ± standard deviation (SD). In the event of failure to follow a normal distribution, the values of TG, FINS, HOMA-IR, and HOMA-*β* were presented as medians (the upper and lower quartiles). Variables that were nonnormally distributed were log-transformed before analysis. Comparisons between the groups were assessed by the independent sample *t*-test and ANOVA test. The differences of proportions were analyzed by the chi-square test. The changes in parameters from baseline within groups were evaluated using the two-tailed paired *t*-test. Multivariate regression analysis was applied to assess the correlations between the change in HbA1c level and relevant variables. Statistical significance was inferred when *P* < 0.05.

## 3. Results

### 3.1. Baseline Characteristics in the Exenatide and Metformin Groups

Table 1 presents the baseline characteristics of the exenatide and metformin groups. Age, gender, the proportion of obese patients, body weight, BMI, TC, LDL-C, HDL-C, TG, FBG, FINS, HbA1c, HOMA-IR, and HOMA-*β* were comparable in the two groups (all *P* > 0.05).

### 3.2. Changes in Anthropometric Measurements after Exenatide or Metformin Treatment

During therapy, 4 patients (3.6%) in the exenatide group dropped out of the study because of moderate-to-severe nausea and vomiting, whereas 2 patients (1.7%) in the metformin group dropped out because of moderate-to-severe vomiting and diarrhea.

After the 12-week treatment, significant reductions in body weight and BMI were observed in the exenatide and metformin groups (all *P* < 0.05), respectively (Table 2). However, the exenatide treatment demonstrated a greater declination in the body weight and BMI as compared to the metformin treatment (all *P* < 0.01) (Table 2).

### 3.3. Alterations in Glucose Metabolic Parameters after Exenatide or Metformin Treatment

Both exenatide and metformin treatments significantly decreased the FBG and HbA1c levels at 12 weeks (all *P* < 0.05) (Table 2); however, no difference was observed in the reductions in FBG between exenatide and metformin treatments. Nevertheless, the HbA1c levels in the exenatide group were reduced more than those in the metformin group [−2.81 (−3.42 to 2.45) versus −1.44 (−1.67 to 1.21), *P* < 0.01] (Table 2). The proportion of patients with HbA1c < 7.0% (53 mmol/mol) in the exenatide group was 79.5%, which was higher than the 64.0% in the metformin group (*P* < 0.05) (Table 2) [3].

We also observed a significant increase in HOMA-*β* in the two groups after exenatide or metformin treatment (all *P* < 0.05) (Table 2). The increase in the HOMA-*β* value of the exenatide group was higher than that of the metformin group [33.01 (20.19–45.82) versus 14.03 (6.34–21.72), *P* < 0.01]. However, the decrease in the HOMA-IR value was similar after 12 weeks of exenatide or metformin treatment (*P* > 0.05) (Table 2).

Interestingly, exenatide did not cause a greater decrease in the HbA1c level than metformin after adjustment for the reduced body weight following the 12-week treatment (*P* > 0.05). However, the greater increase in the HOMA-*β* value was still observed in the exenatide group than in the metformin group postadjustment for the decrease in FBG (*P* < 0.05).

### 3.4. Changes in Lipid Profile after Exenatide or Metformin Treatment

Both exenatide and metformin significantly reduced the TC levels after 12 weeks of treatment (*P* < 0.05) (Table 2). Moreover, a significant decrease in the plasma TG levels of the exenatide group and LDL-C levels of the metformin group was also observed posttreatment (all *P* < 0.05) (Table 2). In the exenatide group, the TG levels showed a greater decline as compared to those in the metformin treatment group (*P* < 0.01) (Table 2).

### 3.5. Multivariate Regression Analysis to Find the Correlation between the Decrease in HbA1c Level and Relevant Variables

Multivariate regression analysis was used to further assess the relationships between the decrease in the HbA1c level and related variables. The influence of six variables such as age, gender, body mass index, baseline HbA1c, baseline FBG, and drug treatment was assessed. The analysis indicated that the exenatide treatment (*β* = 0.41, *P* < 0.01) and baseline HbA1c level (*β* = −0.84, *P* < 0.01) were independent influencing factors for the decrease in HbA1c level.

### 3.6. Adverse Events

During the 12-week treatment with exenatide or metformin, no major hypoglycemia was observed in any patient (Table 3). Incidences of minor hypoglycemia occurred in 5/106 (4.7%) and 3/118 (2.5%) patients treated with exenatide and metformin, respectively (Table 3).

## 4. Discussion

In the present study, both exenatide and metformin significantly decreased the HbA1c levels in overweight/obese patients with newly diagnosed T2D. After the 12-week treatment, a reduction in HbA1c and the proportion of patients with HbA1c < 7.0% (53 mmol/mol) were higher in the exenatide group than in the metformin group. The exenatide treatment caused a greater decline in the body weight and BMI as compared to the metformin treatment. Multivariate regression analysis indicated that the exenatide treatment and baseline HbA1c level were independent influencing factors for the decrease in HbA1c level.

In our study, metformin reduced the FBG levels by 2.17 mmol/L and the HbA1c levels by 1.44% from baseline, which was consistent with previous studies [9, 10]. However, in the exenatide group, the mean changes in FBG and HbA1c from baseline were −2.62 mmol/L and −2.81%, respectively, higher than the decrease in other studies [4–6, 10]. One possible explanation is that our study assessed the initial therapy of exenatide, and the previous studies mainly evaluated exenatide as the add-on treatment in patients with suboptimum glycemic control after the initial therapy [4–6, 10]. As the preliminary treatment, the improvement of metabolic disorders is associated with the effect of exenatide and lifestyle modification [11]. Moreover, overweight/obese patients with T2D might present a better lifestyle modification if treated with exenatide, which can cause the slowing of gastric emptying and suppression of appetite [12]. Another reason might be associated with the differences in the race [13]. Western population with T2D showed a significant reduction in the GLP-1 sensitivity; however, no significant reduction was seen in Asians with T2D [14, 15]. Thus, the preserved GLP-1 sensitivity might account for the greater glucose-lowering efficacy of exenatide treatment in our study [14, 15]. In addition, Chinese T2D patients have higher postprandial glucose levels than Caucasians, and exenatide has a greater effect on postprandial glucose by stimulating glucose-dependent insulin secretion, inhibiting postprandial glucagon, and delaying the gastric discharging [12, 16]. In our study, exenatide treatment resulted in a greater reduction in HbA1c levels than metformin treatment. The proportion of patients with HbA1c < 7.0% (53 mmol/mol) in the exenatide group was 79.5%, which was higher than the 64.0% in the metformin group. Multivariate regression analysis indicated that the exenatide treatment was an independent influencing factor for the decrease in HbA1c level. These results are consistent with those of a meta-analysis, which demonstrated that GLP-1 receptor agonists contribute to a larger proportion of patients achieving HbA1c < 7.0% (53 mmol/mol) than metformin [17]. Thus, the results might suggest that for initial therapy in overweight/obese patients with newly diagnosed T2D, exenatide causes a better glycemic control than metformin.

Obesity is associated with T2D by inducing endoplasmic reticulum stress, chronic inflammatory state, and insulin resistance [2]. Our study demonstrated that exenatide and metformin individually caused a significant decrease in body weight. A greater weight loss was observed in the exenatide group compared to the metformin group. These results for metformin are consistent with those of our previous and other studies [9, 18]. Metformin leads to a moderate weight loss by several mechanisms, including increasing insulin sensitivity, decreasing gastrointestinal absorption of carbohydrates, and reducing ghrelin and leptin levels after glucose overload [19]. In addition, metformin increased circulating levels of active forms of GLP-1, which might be involved in the suppression of appetite [20]. In the present study, exenatide significantly decreased the body weight by 5.79 kg. Exenatide lowers body weight by slowing gastric emptying, promoting satiety, and decreasing food intake [12]. In addition, lifestyle intervention, especially diet control, can also effectively reduce the body weight in patients with T2D [21]. Due to the suppression of appetite, GLP-1 receptor agonist might exhibit a synergistic effect with life intervention and contribute to improved diet control and hence more weight loss. Notably, after adjustment for the decrease in body weight, no significant difference was observed in the reduction in HbA1c between the exenatide and metformin treatments. This suggested that the better hypoglycemic effect of exenatide on overweight/obese patients might be primarily associated with enhanced weight loss.

Insulin resistance plays a major role in the pathogenesis of T2D and its complications [2]. Metformin has been widely established as a strong insulin sensitizer [3]. The present study showed that exenatide and metformin similarly decreased insulin resistance in overweight/obese patients with T2D. The significant reduction in body weight is one of the key reasons for the improvement of insulin resistance of exenatide and metformin [22]. Both GLP-1 receptor agonist and metformin exerted some beneficial effects on insulin resistance indirectly by inhibiting oxidative stress and regulating lipid metabolism [23, 24]. Thus, it might suggest that exenatide is another insulin sensitizer that leads to amelioration of insulin resistance in obese patients with T2D.

Compared to the Western population, Asians with T2D showed a distinct *β*-cell dysfunction [25]. In the present study, both exenatide and metformin treatments significantly increased HOMA-*β* values, and the increase in the exenatide group was higher than that in the metformin group. The alleviation of glucose toxicity, rather than a direct drug effect, might underlie the HOMA-*β* improvement in the two groups [26]. However, after adjustment for the decrease in FBG, the increase in the HOMA-*β* value was still observed in the exenatide group than in the metformin group. Previous studies showed that metformin indirectly increased *β*-cell function through decreasing glucotoxicity, lowering insulin resistance, inhibiting the formation of advanced glycation end products, and reducing oxidative stress [23, 26]. In addition to the similar capabilities of metformin, exenatide also displays some direct beneficial effects on islet *β*-cells [27–30]. A previous study showed that exenatide improved the insulin secretion of *β*-cells as compared to the insulin glargine in a similar glycemic control, and this effect was sustained after a 4-week off-drug period [28]. Animal studies have demonstrated that GLP-1 promotes the proliferation of *β*-cells and inhibits their apoptosis [29, 30]. Cell-based studies showed that GLP-1 enhanced the viability and inhibited the mitochondrial-dependent apoptosis in the PKC-dependent pathway [30].

Notably, our study has several limitations. The major limitation was that the present study was not a randomized controlled trial. In addition, our study also lacked accurate methods, such as hyperglycemic and hyperinsulinemic clamp techniques, to assess *β*-cell function and insulin sensitivity. Furthermore, the treatment duration was relatively short. Long-term observation of these treatments is needed to evaluate the persistence of beneficial effects over time. Moreover, the cost/benefit ratio is another aspect that should be considered. In terms of medical expenses, metformin might be more preferable for a lifelong treatment. Despite these limitations, one implication of the present study is that for a patient with newly diagnosed T2D, exenatide might contribute to a better effect on glycemic control, weight management, and *β*-cell function, which at least partly represents an avenue for investigation in the treatment of T2D.

In conclusion, for an initial therapy in overweight/obese patients with newly diagnosed T2D, exenatide causes a better glycemic control than metformin, whose mechanism might potentially be related to the better weight loss effect and amelioration of *β*-cell function.

## Figures and Tables

**Table 1:** Baseline characteristics of the exenatide and metformin groups.

Parameters	Exenatide (*n* = 110)	Metformin (*n* = 120)	*P*
Age, y	45.77 ± 10.60	48.05 ± 8.04	0.097
Gender, males/females, *n*	64/46	71/49	0.880
Overweight/obesity, *n*	57/53	63/57	0.918
Body weight, kg	78.76 ± 10.01	77.98 ± 11.88	0.907
BMI, kg/m^2^	27.83 ± 2.38	27.57 ± 3.56	0.815
TC, mmol/L	5.21 ± 1.25	5.29 ± 1.22	0.664
LDL-C, mmol/L	3.07 ± 0.89	3.05 ± 0.90	0.864
HDL-C, mmol/L	1.12 ± 0.43	1.24 ± 0.29	0.087
TG, mmol/L	2.07 (1.27–3.99)	1.93 (1.32–2.75)	0.235
FBG, mmol/L	9.34 ± 3.27	9.10 ± 1.34	0.594
FINS, *μ*IU/mL	9.50 (5.98–14.37)	10.14 (5.25–14.94)	0.337
HbA1c, %	9.11 ± 1.46	9.03 ± 1.18	0.230
HOMA-IR	3.94 (2.47–5.93)	3.87 (2.27–6.02)	0.638
HOMA-*β*	55.13 (29.64–76.65)	50.55 (29.80–74.81)	0.289

Data are means ± SD unless indicated otherwise. TG, FINS, HOMA-IR, and HOMA-*β* are shown as medians (upper and lower quartiles). BMI: body mass index; TC: total cholesterol; LDL-C: low-density lipoprotein cholesterol; HDL-C: high-density lipoprotein cholesterol; TG: triglyceride; FBG: fasting blood glucose; FINS: fasting insulin; HOMA-IR: homeostasis model assessment of insulin resistance; HOMA-*β*: homeostasis model assessment of *β*-cell function.

**Table 2:** Changes in metabolic parameters after metformin or exenatide treatment.

Parameters	Exenatide (*n* = 106)	Metformin (*n* = 118)	*P*
Body weight, kg	−5.79 (−8.09 to −3.48)	−2.30 (−0.99 to −0.66)	0.001
BMI, kg/m^2^	−1.99 (−2.85 to −1.14)	−0.83 (−0.99 to −0.66)	0.000
TC, mmol/L	−0.58 (−1.00 to −0.15)	−0.35 (−0.52 to −0.19)	0.252
LDL-C, mmol/L	−0.17 (−0.42 to 0.08)	−0.14 (−0.26 to −0.03)	0.900
HDL-C, mmol/L	0.06 (−0.02 to 0.13)	−0.04 (−0.08 to 0.00)	0.358
TG, mmol/L	−1.26 (−2.07 to −0.45)	−0.03 (−0.31 to 0.26)	0.000
FBG, mmol/L	−2.62 (−3.89 to −1.35)	−2.17 (−2.51to −1.73)	0.321
FINS, *μ*IU/mL	0.63 (−1.19 to 2.46)	−2.03 (−4.50 to −1.52)	0.000
HbA1c, %	−2.81 (−3.42 to −2.45)	−1.44 (−1.67 to −1.21)	0.000
HOMA-IR	−2.05 (−2.69 to −1.37)	−1.88 (−2.23 to −1.06)	0.565
HOMA-*β*	33.01 (20.19–45.82)	14.03 (6.34–21.72)	0.002
HbA1C < 7.0%, *n* (%)	84 (79.5)	75 (64.0)	0.033

Data are shown as difference (95% CI) versus baseline, except for HbA1C < 7.0%. BMI: body mass index; TC: total cholesterol; LDL-C: low-density lipoprotein cholesterol; HDL-C: high-density lipoprotein cholesterol; TG: triglyceride; FBG: fasting blood glucose; FINS: fasting insulin; HOMA-IR: homeostasis model assessment of insulin resistance; HOMA-*β*: homeostasis model assessment of *β*-cell function; HbA1C < 7.0%: the proportion of patients with HbA1c < 7.0% (53 mmol/mol).

**Table 3:** Adverse events in the exenatide and metformin groups.

Adverse events	Exenatide (*n* = 106)	Metformin (*n* = 118)	*P*
Major hypoglycemia, *n* (%)	0	0	1
Minor hypoglycemia, *n* (%)	5 (4.7%)	3 (2.5%)	0.481
